# Recurrent Miller Fisher Syndrome

**DOI:** 10.7759/cureus.26192

**Published:** 2022-06-22

**Authors:** Say Ting Ooi, Ameilia Ahmad, Azhany Yaakub

**Affiliations:** 1 Department of Ophthalmology and Visual Science, School of Medical Sciences, Universiti Sains Malaysia, Kota Bharu, MYS; 2 Ophthalmology Clinic, Hospital Sultanah Nur Zahirah, Kuala Terengganu, MYS

**Keywords:** guillain-barré syndrome, ataxia, areflexia, ophthalmoplegia, miller fisher syndrome

## Abstract

Miller Fisher syndrome (MFS) is an uncommon systemic autoimmune condition. Recurrent Miller Fisher syndrome is extremely rare. We want to highlight a rare case of recurrent Miller Fisher syndrome, which manifested as external and internal ophthalmoplegia, areflexia, and ataxia following an episode of upper respiratory tract infection (URTI). The patient developed a recurrent attack of Miller Fisher syndrome two months later with only internal and external ophthalmoplegia symptoms. Both episodes wholly resolved in a month without treatment. Miller Fisher syndrome can mimic various other neurological illnesses. Therefore, diagnosing this disease is often challenging. However, prompt diagnosis and management can be achieved with awareness of this rare illness.

## Introduction

Miller Fisher syndrome (MFS) is a rare autoimmune neurological disease. Charles Miller Fisher identified MFS as a subset of Guillain-Barré syndrome (GBS) in 1956 [1]. It has a worldwide prevalence of one in 1,000,000 [2]. The percentage of MFS in GBS is higher among Asian than in Western populations. It accounts for approximately 5% of GBS in the Western population, while it ranges between 17% and 25% in the Asian population [2-4].

In 1932, James Collier first defined MFS as a triad of ophthalmoplegia, ataxia, and areflexia. MFS usually has a monophasic presentation and a good recovery prognosis; recurrent disease is rare [5,6]. We want to highlight a recurrent case of MFS in a young female in which the symptoms wholly resolved spontaneously.

Part of this article was previously presented as a poster at the 35th Singapore-Malaysia Joint Meeting in Ophthalmology on January 17-19, 2020, in Singapore.

## Case presentation

A 19-year-old female without any medical illness experienced binocular diplopia and unsteady gait. It was preceded by an upper respiratory tract infection (URTI) with fever. On day 5 of URTI, she developed binocular diplopia. It was associated with bilateral eye photophobia, blurring of vision, mild retro-orbital pain on eye movement, and unsteady gait. There was no symptom of high intracranial pressure.

**Figure 1:**
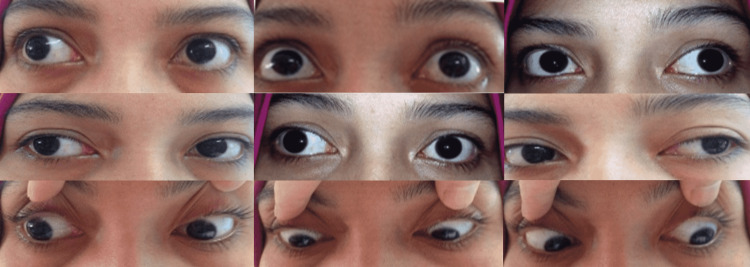
Bilateral external ophthalmoplegia with variable weakness on all gazes.

**Figure 2:**
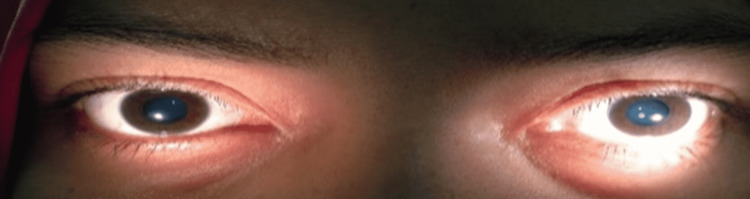
Bilateral internal ophthalmoplegia with dilated pupil.

Upon examination, her bilateral vision was 6/18 with no improvement on pinhole. The relative afferent pupillary defect was equivocal. Both eyes demonstrated external and internal ophthalmoplegia. Bilateral ocular movement showed variable weakness on all gazes (Figure 1). Both eyes’ pupils were sluggish and dilated at 5 mm (Figure 2). Other anterior and posterior segment examinations were normal. Other cranial nerve examinations were normal, particularly no bulbar anomalies. All upper and lower limb power was 4/5 with areflexia. She had a broad-based gait with proprioception loss. Cerebellar signs were negative.

Her urine toxicology test and brain CT were normal. Cerebrospinal fluid (CSF) electrophoresis showed increased protein (0.88) and albumin levels (495); no oligoclonal band was noted. Serum anti-GQ1b IgM, IgG, and anti-GT1a IgG were detected. *Mycoplasma pneumoniae* serology was also positive (1:1280). Based on the clinical findings and laboratory investigation results, Miller Fisher syndrome was diagnosed.

**Figure 3:**
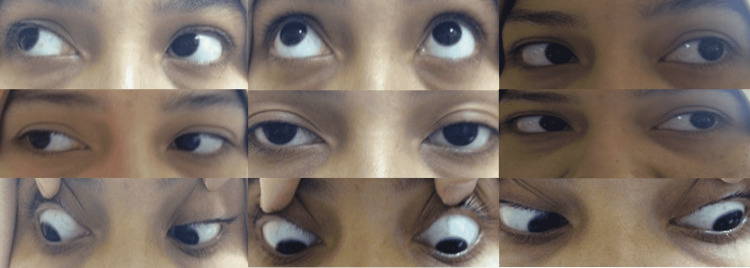
Complete recovery of external ophthalmoplegia.

**Figure 4:**
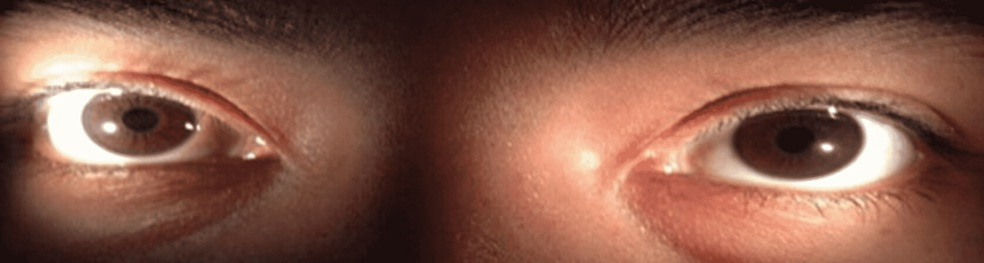
Complete recovery of internal ophthalmoplegia.

She was treated conservatively, and she began to show signs of improvement a few days later in the sequence of lower limb weakness, ataxia, internal and external ophthalmoplegia, and then areflexia. Full recovery was achieved in one month (Figures 3, 4).

Two months after the first MFS attack, she developed a recurrent disease following brief URTI symptoms. During this episode, examination only showed external and internal ophthalmoplegia. Other ophthalmological and neurological examinations were normal; there was no ataxic gait or areflexia. She was again treated conservatively, and her condition improved completely about a month later.

## Discussion

MFS is a rare autoimmune disorder. It is caused by a cross-reaction of anti-GQ1b antibody against gangliosides following infections caused by *Campylobacter jejuni*, *Mycoplasma pneumoniae*, and *Haemophilus influenzae*, which typically manifests as URTI or gastroenteritis. MFS is difficult to diagnose as the presenting symptoms can mimic various common medical problems such as myasthenia gravis, drug intoxication, brainstem strokes, and botulism. MFS is usually presented as a triad of ophthalmoplegia, ataxia, and areflexia. Ophthalmoplegia is typically bilateral and symmetrical [7]. Both internal and external ophthalmoplegia is common [4,7]. Although areflexia was included in the triad, it was less common than the other two symptoms [4,7]. Other GBS-related symptoms that may also appear in MFS include limb weakness; loss of proprioception, sensation, and vibration; bulbar palsy that manifests in the pharyngeal-cervical-brachial (PCB) variant of GBS; reduced consciousness and pyramidal sign in Bickerstaff brainstem encephalitis (BBE); and autonomic disturbance such as urinary retention, arrhythmia, and hypotension. During the first episode of our patient, she presented with MFS/GBS overlap syndrome that manifested with MFS triad, acute limb weakness, and loss of proprioception [4,7].

MFS is a clinical diagnosis. Since the discovery of the anti-GQ1b antibody, detection of MFS has become easier. However, extreme caution is advisable as the antibody is also tested positive in the potentially lethal GBS variants such as BBE and PCB [7,8]. Serum anti-GQ1b antibody outperforms cerebrospinal fluid (CSF) anti-GQ1b antibody in identifying MFS [9]. The sensitivity and specificity of serum anti-GQ1b antibody are both high, at 92% and 97%, respectively [9]. CSF anti-GQ1b has a 100% specificity but only 20% sensitivity [9]. Anti-GT1a antibody frequently coexists with anti-GQ1b antibody. They often manifest clinically as MFS [9,10]. However, similar to the anti-GQ1b antibody, the anti-GT1a antibody was positive for other GBS variants, particularly PCB [10]. CSF analysis is necessary to rule out other potential disorders such as meningitis or multiple sclerosis. In CSF studies, MFS is characterized by elevated albumin or protein levels with a normal cell count [2,7].

Recurrent MFS is rare, accounting for 11%-14% of all MFS cases [5,6]. Younger people are more likely to experience recurrent MFS [5]. Recurrent disease can appear early and have numerous episodes [5,6]. The clinical features of recurrent MFS are identical to those of nonrecurrent MFS [6]. However, the severity of the disease can vary. In our case, the severity of recurrent MFS was milder. Only acute internal and external ophthalmoplegia was seen in the patient. Because ophthalmoplegia has the strongest association with MFS, it is the only symptom that manifests during mild MFS [5,8]. Genetic involvement in recurrent MFS is still unclear. Some studies found that patients with recurrent MFS tested positive for HLA-DR2 [6]. Therefore, genetic factors may play a role in recurrent MFS.

The treatment of MFS is still controversial between conservative and active management. Options of active treatment included plasmapheresis and intravenous immunoglobulin (IVIG). It is recommended that active management be considered in severe and life-threatening cases such as respiratory depression, MFS/PCB overlap syndrome, Bickerstaff brainstem encephalitis, and MFS/GBS overlap syndrome [11]. IVIG can hasten the recovery of ophthalmoplegia and ataxia, but it does not alter the outcome. This is likely due to the good natural prognosis of the disease [4,11]. Recovery of the syndrome often follows the sequence of ataxia, ophthalmoplegia, and finally, areflexia [4]. In this case, the patient gained rapid spontaneous recovery in both MFS attacks. Hence, IVIG was not given. The benefits of IVIG in preventing illness recurrence were uncertain. More clinical trials will be required to improve the understanding of the effects of IVIG on recurrent MFS.

## Conclusions

MFS is a rare neurological condition. It is often misdiagnosed as other more common but life-threatening neurological conditions such as drug intoxication, brainstem strokes, botulism, and myasthenia gravis. However, awareness and knowledge of the disease can help clinicians in making accurate diagnoses and management decisions. Although MFS generally has a good prognosis and achieves spontaneous recovery, clinicians should know that potentially life-threatening conditions still can happen in MFS. Therefore, a thorough clinical assessment is vital in managing MFS.
